# Comparative Evaluation of Transient Protein Expression Efficiency in Tissues across Soybean Varieties Using the Tsukuba System

**DOI:** 10.3390/plants13060858

**Published:** 2024-03-16

**Authors:** Martina Bianca Fuhrmann-Aoyagi, Saki Igarashi, Kenji Miura

**Affiliations:** 1 Graduate School of Science and Technology, University of Tsukuba, Tsukuba 305-8572, Japan; s2136022@u.tsukuba.ac.jp (M.B.F.-A.); s2012074@s.tsukuba.ac.jp (S.I.); 2 Tsukuba-Plant Innovation Research Center, University of Tsukuba, Tsukuba 305-8572, Japan

**Keywords:** *Glycine max*, GFP, agroinfiltration

## Abstract

Transient protein expression is a versatile tool with diverse applications and can be used in soybeans to study gene function, obtain mutants, and produce proteins for commercial use. However, soybeans are considered recalcitrant for agroinfiltration. Subsequent studies on soybeans have demonstrated a green fluorescent protein (GFP) expression in seedpods, but not in leaves, using syringe agroinfiltration. To evaluate agroinfiltration-based transient protein expression levels in plant cells, we used the transient expression vector pTKB3 harboring the *GFP* gene. Using *Agrobacterium tumefaciens*, vacuum agroinfiltration of the leaves and needle agroinfiltration of the seedlings of different soybean varieties were performed. GFP was transiently expressed in all of the samples. However, the Enrei and Williams 82 varieties presented better results than the other varieties in the leaf tissue, with results confirmed by immunoblot analysis, demonstrating that both varieties are good candidates for molecular biological studies. GFP expression in the seedlings was less extensive than that in the leaves, which may be due to the tissue characteristics, with Enrei showing the best results. Based on this observation, we conclude that the Tsukuba system is an effective tool that can be used for different tissues and soybean varieties.

## 1. Introduction

Soybean (*Glycine max*) is a globally important crop as it is a source of protein and oil for food, feed, and biofuels [1]. It is a model plant of the legume family and its entire genome has been sequenced [2]. The results obtained from soybean studies can be easily replicated in other leguminous species, such as *Medicago truncatula*, owing to their genome similarity [3]. Moreover, it has attracted attention as a host plant for biopharmaceutical production owing to its low production cost, high protein content, and long shelf life [4,5].

The Tsukuba system is a transient protein expression system in plants that combines a geminivirus-derived rolling cycle replication system and a double terminator [6], allowing for transient protein expression without creating transformants, thus reducing the required time. Silencing and other repression mechanisms are occasionally activated when target proteins are expressed using transformants. However, these mechanisms are not well activated during transient expression, enabling the expression of large amounts of proteins [6,7]. Furthermore, the Tsukuba system can be used to obtain mutants when combined with CRISPR-Cas technology [8]. However, studies on transient protein expression in soybean are limited, and this species is considered recalcitrant to transformations.

A transient expression by agroinfiltration has been demonstrated in some legume species, including the common bean (*Phaseolus vulgaris*) [9,10], *Medicago truncatula* [11], cowpea (*Vigna unguiculata*) [12], lotus (*Lotus japonicus*) [10,13], pea (*Pisum sativum*) [14,15], and *Caragana intermedia* [16]. Transient protein expression has been reported in soybean pods [10], protoplasts [17], and roots [18]. A previous study [10] has reported challenges in achieving consistent expression, particularly in intact tissues such as leaves, because the characteristics of soybean cells pose unique obstacles. Understanding the dynamics of transient protein expression in soybean is pivotal because of its perceived recalcitrance to transformations. By examining the intricacies of transient expression across different soybean varieties and tissues, this study aimed to unravel the underlying factors contributing to the recalcitrant nature of soybeans. Such insights could pave the way for targeted improvements in transformation methodologies, potentially overcoming the barriers that hinder efficient gene and protein expression in soybeans.

## 2. Results

### 2.1. Microscopic Analysis of Green Fluorescent Protein Expression

**Figure 1:**
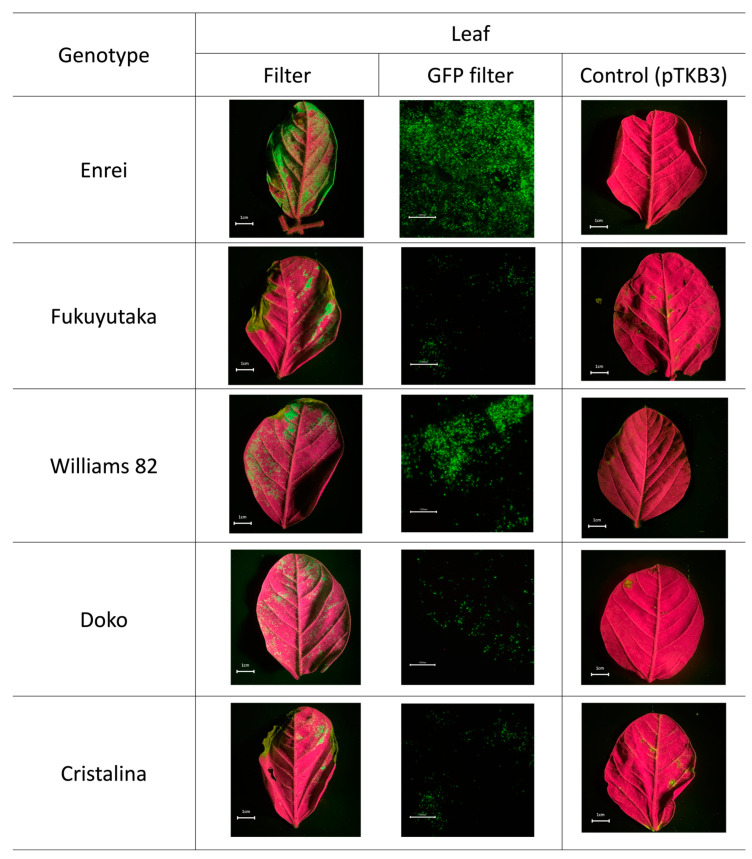
Green fluorescent protein (GFP) expression in plant leaves. Images depict GFP expression in leaves, showing the fluorescence captured by the filter (Fujifilm SC-52) and camera, followed by the detailed GFP expression observed through the microscope. The last image compares the filtered photos of the control samples, emphasizing the specificity of the fluorescence in relation to the experiments. The white bar in the filter picture indicates 1 cm in length. The bar in the microscope picture represents 0.1 cm in length.

Green fluorescent protein (GFP) expression occurred widely in >80% Enrei leaf tissues. A high transient GFP expression in the leaves of the Enrei variety was confirmed by microscopy. In addition to the Enrei variety, the Williams 82 variety, which is commonly used in genetic transformation studies, also exhibited a notable GFP expression. Although the coverage of the leaf area was not extensive, a high expression was observed in the microscopic images. Conversely, the other varieties, Fukuyutaka, Doko, and Cristalina, showed a less significant expression, both in the filter and microscope photos. Moreover, GFP was not expressed in the samples infiltrated solely with the pTKB3 plasmid (Figure 1). To determine whether the high GFP expression occurred because of the infiltration buffer or the technique itself, Enrei leaves were infiltrated using a syringe. However, punctual GFP expression was observed using a microscope only at points near where the syringe infiltration was performed (Figure S1). The GFP expression in the leaves was detected starting from day 2 post-infiltration and persisted until day 10 (Figure S2).

**Figure 2:**
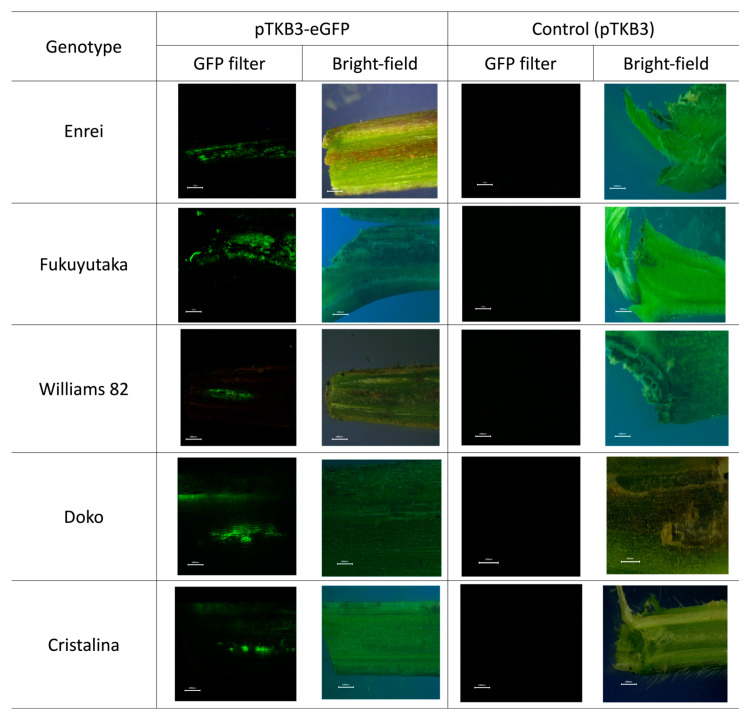
Green fluorescent protein (GFP) expression in seedlings. Images depict the GFP expression in seedlings, including detailed micrographs obtained using a bright field microscope. The last image compares the photos of the control samples, emphasizing the specificity of the fluorescence in relation to the experiments. The white line represents 0.1 cm in length.

The expression area for seedling samples was calculated based on the syringe needle size (approximately 0.5 cm). The GFP expression was homogeneous and comprehensive in the Enrei and Fukuyutaka varieties. The Williams 82, Doko, and Cristalina varieties showed a punctual expression in the stem tissue. GFP was not expressed in The samples infiltrated with only the pTKB3 plasmid (Figure 2).

**Figure 3:**
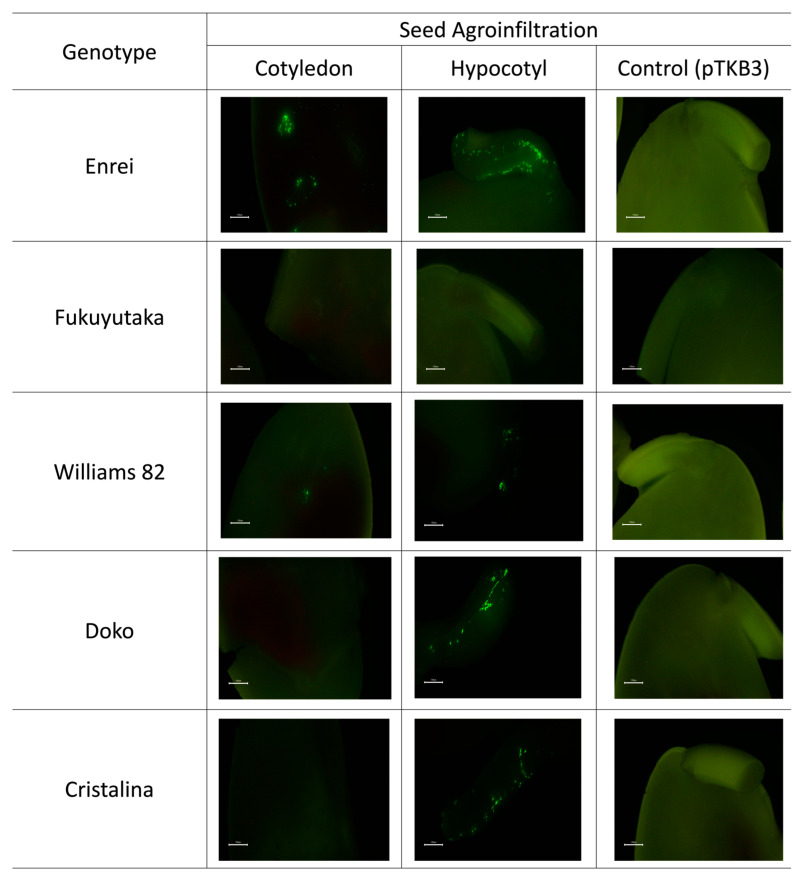
Green fluorescent protein (GFP) expression in seeds. Images depict GFP expression in seedlings, including detailed micrographs obtained using a bright field microscope. The first and second image demonstrate different tissues in soybean seeds. The last image compares the photos of the control samples, emphasizing the specificity of the fluorescence in relation to the experiments. The white line represents 0.1 cm in length.

The seed cotyledon and hypocotyl were assessed for GFP expression. Both tissues exhibited a GFP expression in the Enrei and Williams 82 varieties, with Enrei displaying a wider expression area compared with Williams 82. However, the Doko and Cristalina varieties showed expression exclusively in the hypocotyl. In contrast, no GFP expression was observed in either tissue for the Fukuyutaka variety. Additionally, seeds infiltrated solely with the pTKB3 plasmid did not exhibit a GFP expression (Figure 3).

### 2.2. Comparative GFP Expression Levels across Soybean Varieties

From the collected images, the expression levels were represented and classified as medium to high expression when they covered more than 50% tissue, and as medium to low expression when they covered less than 50% tissue. Interestingly, only the Enrei variety showed a high expression in both tissues (Table 1). This distinction highlights the notable differential response of this variety compared with that of the other varieties in the context of the gene expression in the tissues analyzed.

### 2.3. Protein Quantification

**Figure 4:**
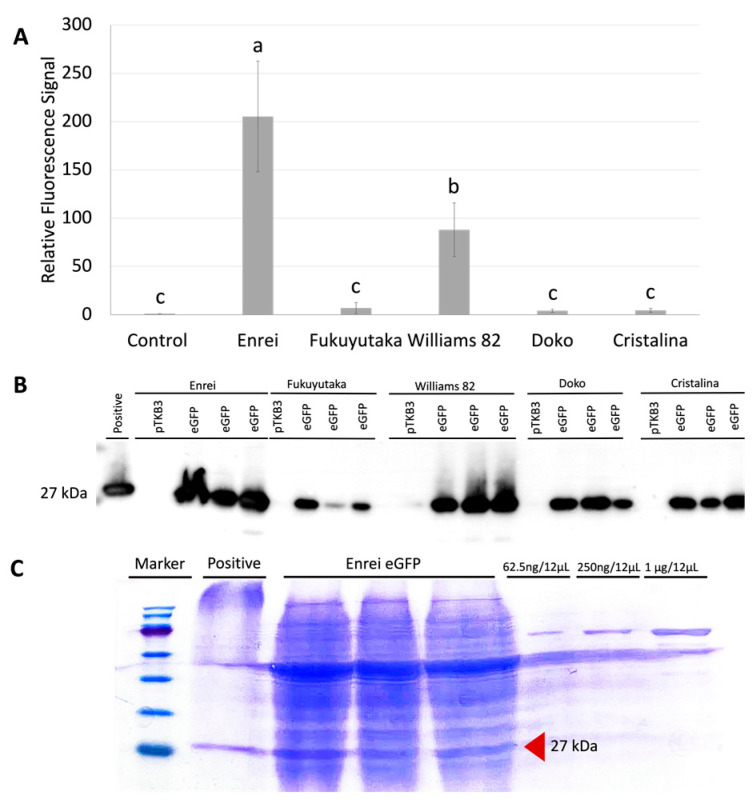
Analysis of the green fluorescent protein (GFP) expression in the leaf samples of different soybean varieties. (**A**) Graphical representation of the relative fluorescence signal expression, highlighting the variations in the GFP signal intensity between the Enrei, Fukuyutaka, Williams 82, Doko, and Cristalina samples, and a comparison with control samples. Mean values ± SD for *n* = 3. Different lowercase means significant differences in the Tukey’s test (*p* < 0.05). (**B**) Western blot analysis of the soybean samples including a positive control (lettuce samples expressing pTKB3-eGFP plasmid) with an anti-GFP antibody and a marker for reference. All of the samples were diluted 40 times the initial concentration. (**C**) Gel stained with Coomassie Brilliant Blue (CBB) to compare the total amount of protein in the Enrei leaf samples and different bovine serum albumin (BSA) concentrations for comparison. Red arrow indicates the GFP protein size.

An efficient GFP expression in soybean tissues after *A. tumefaciens* infiltration was observed under a microscope. After quantifying the expression intensity, the leaf samples from Enrei, Williams 82, Fukuyutaka, Doko, and Cristalina varieties presented a 205-, 88-, 6.9-, 4.1-, and 4.5-fold increase in expression compared with the control leaf samples, respectively (Figure 4A). These results were confirmed by immunoblot analysis (Figure 4B), where the GFP expression was detected only in the pTKB3-eGFP-infiltrated samples for all of the varieties. This signal was extremely intense in the Enrei and Williams 82 varieties, confirming the relative fluorescence signal data. As the Enrei variety exhibited the best expression among the samples, the protein quantification method per mg fresh weight was used only for the same samples; based on the gel, the Enrei leaf samples had approximately 0.6 μg/mg of fresh mass (Figure 4C).

## 3. Discussion

In this study, we demonstrated the transient green fluorescent protein (GFP) expression in soybean leaves and seedlings. Soybean is considered a recalcitrant species for transformation and other procedures [19,20], and few studies have explored the transient expression in intact soybean tissues. To date, research has generally focused on a stable GFP expression through tissue culture transformation, rather than in intact tissues. A previous study did not observe GFP expression in the leaves of the Enrei and Fukuyutaka soybean varieties, although a significant GFP expression was observed in the pods [10]. Remarkably, GFP expression persisted in the leaves for a duration of 10 days. This duration of transient expression aligned with the findings from a previous study, which similarly demonstrated GFP expression lasting for 10 days in tobacco leaves [21]. The agroinfiltration methodologies used in the previous and present studies were different. Previously, syringe agroinfiltration was conducted on the leaves, while agroinfiltration was performed in this study using the vacuum technique. The GFP expression after syringe-based agroinfiltration was low and the GFP fluorescence emission was detected only through microscopic analysis (Figure S1). Therefore, vacuum agroinfiltration is suitable for transient expression in soybean leaves. The leaf samples were vacuum-infiltrated three times for 5 min each, with a break between the vacuum treatments. These deliberate adjustments may be the key to improving the cellular permeability and potentially influencing the GFP expression outcomes. A modified infiltration buffer was used for this study. L-cysteine, dithiothreitol, and/or sodium thiosulfate may enhance the transient GFP expression in leaves. The expression was also the highest in the Enrei leaves and seedlings, indicating that this variety is extremely susceptible to infection by *Agrobacterium*. It has been used in several studies in Japan as a variety less recalcitrant to transformation processes, as demonstrated by CRISPR-Cas9 and other molecular biological techniques [22,23,24]. Its genome has also been sequenced [22]. King et al. [25] reported expression differences among varieties, where the Peeking and Williams 82 varieties presented excellent expression results. The differences between the varieties may be due to defense mechanisms against infections present in soybeans; however, future RNA-seq studies are necessary to compare these genes between varieties.

The expression in seedlings was not as widely distributed as that in the leaves. This limitation can be attributed to the intrinsic characteristics of stem cells, as the leaves contain a large amount of parenchyma. In contrast, the stem consists of various cells with different amounts of collenchyma and sclerenchyma [26,27,28]. Additionally, using a needle imposes restrictions on the amount of infiltration solution that can be introduced. Moreover, even when vacuum agroinfiltration is performed on the entire plant, observing the expression in the petioles and stem is often not possible [29,30]. However, studies focused on the seedling expression have potential relevance for future in planta transformation procedures [31,32]. An example of this potential was demonstrated in a study by Hu et al. [33] with radishes, who obtained mutants through an in planta transformation procedure in seedlings. These results suggest that despite the limitations observed in the expression distribution in seedlings, these experiments may provide valuable insights into the development of in planta transformation techniques in the future. An in-depth understanding of these processes at different plant development stages is critical for effective and comprehensive advances in plant genetic engineering. For seed transformation, the Tsukuba system demonstrated a significant expression in cotyledon and hypocotyl tissues, as illustrated by the findings of the GFP expression in these tissues, as reported by Benzle et al. [34]. The study also suggests that the expression levels may vary depending on the *A. tumefaciens* strains utilized. Additionally, further adjustments, such as altering the incubation time and *A. tumefaciens* concentration, could be explored in future studies to enhance the expression in seed tissues. These enhancements could potentially establish the Tsukuba System as a robust mechanism for transforming soybean seeds.

In summary, our findings highlight that the Enrei variety is a promising choice for studies on protein expression and transformation. Its robust expression, extensive tissue coverage, and availability of a sequenced genome make it a valuable candidate for further research. Additionally, while the Williams 82 variety, a recognized model, exhibited favorable expression results, Enrei surpassed Fukuyutaka, Doko, and Cristalina in performance, emphasizing its suitability for advanced investigations in molecular biology and genetic studies. Furthermore, the Tsukuba system demonstrated an excellent transient expression in different tissues, potentially expanding its applicability to soybeans and other legumes.

## 4. Materials and Methods

### 4.1. Plant Materials and Growth Conditions

Five commercial soybean varieties, Cristalina, Doko, Enrei, Fukuyutaka, and Williams 82, were used in this study. The seeds were surface-sterilized with 75% ethanol before performing the experiments and were germinated in a growth chamber at 26 °C and a 16 h light/8 h dark photoperiod. After germination, healthy and uniform seedlings were transferred to plastic pots (Prestera 105) filled with soil and maintained under the same conditions until the experiment was completed. During this period, the plants were constantly irrigated and provided with proper nutrients.

### 4.2. Agrobacterium Preparation

To express GFP, pTKB3–eGFP was used, as described previously [7]. For the control samples, the pTKB3 plasmid without GFP was used. The vector was introduced into the *A. tumefaciens* strain GV3101 by electroporation.

### 4.3. Infiltration Procedure and GFP Expression Verification in Agro-Infiltrated Plants

**Figure 5:**
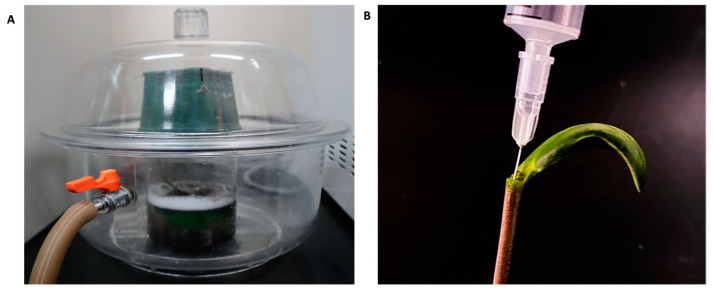
Agroinfiltration procedure. (**A**) Vacuum procedure for leaf infiltration involves positioning the plant upside down and with the leaves in contact with the infiltration buffer. (**B**) The syringe procedure for seedling infiltration includes removing one cotyledon and in the remaining meristem, after the needle is inserted in the stem and the *Agrobacterium tumefaciens* with infiltration buffer is pushed inside the seedling stem.

*A. tumefaciens* carrying the plasmid were initially grown at 28 °C in 5 mL LB medium supplemented with 50 mg/L kanamycin and 30 mg/L rifampicin for 2 days before 1 mL aliquots were transferred in to 200 mL same medium and cultured overnight at 28 °C. Subsequently, OD_600_ was adjusted to 1.0 and the *A. tumefaciens* cells were centrifuged at 4500× *g* for 15 min and resuspended in an infiltration buffer containing 10 mM MgCl_2_, 10 mM 2-(*N*-morpholino) ethanesulfonic acid (MES; pH 5.6), 200 μM acetosyringone, 800 mg/L L-cysteine, 1 mM dithiothreitol, and 1 mM sodium thiosulfate. Three replicates were used for each sample. For leaf infiltration, 21-day-old plants were placed in an infiltration buffer inside a vacuum chamber. The vacuum was applied for 5 min and repeated three times, as described previously [25] (Figure 5A). Additionally, the abaxial part of the Enrei leaves were infiltrated with the same buffer by pressurizing with a syringe. Moreover, 7-day-old seedlings were infiltrated with the buffer with *A. tumefaciens* using a syringe with a 31G needle (0.26 mm × 12 mm) (Figure 5B). The syringe needle was fully inserted into the stem, and the buffer was placed inside. This procedure was repeated three times for each seedling. The plants were kept in the growth chambers at 26 °C with a 16 h light/8 h dark photoperiod. After 3 days, GFP fluorescence was detected and captured using a stereo-fluorescence microscope (MZ FL III; Leica Microsystems, Tokyo, Japan). Emissions from the leaves were captured using a regular digital camera with an SC-52 ultraviolet-absorbing filter.

### 4.4. Agrobacterium-Mediated Seed Transformation

For the seed transformation, the protocol of Paz et al. [35] was used with slight modifications. The cotyledon was divided in half, after the hypocotyl was not completely removed, and the tip of the radicle was cut; then, the seeds were incubated in the solution described in the protocol with *A. tumefacins,* with the OD_600_ adjusted to 1.0 for 10 min. The seeds were transferred to cocultivation medium with filter paper and incubated in growth chambers at 26 °C at a 16 h light/8 h dark photoperiod for 3 days. For each variety, 6 seeds were transformed. The GFP expression was verified, as described above.

### 4.5. Protein Expression Evaluation

Soluble proteins were extracted from leaf samples by crushing and diluting in a lysis buffer, as previously described [36]. To quantify the GFP in each sample, 50 μL of the sample was analyzed using the Varioskan LUX Multimode Microplate Reader (Thermo Fisher Scientific, Waltham, MA, USA) to detect the GFP signal. Western blot analysis was performed using an anti-GFP antibody to confirm the protein amount. A portion of the supernatant was boiled and combined with the sodium dodecyl sulfate (SDS) buffer for 5 min at 95 °C. The samples were loaded on SDS-polyacrylamide gels. After electrophoresis, the proteins were transferred to a PVDF membrane, probed with an anti-GFP monoclonal antibody (GF28R, Thermo Fisher Scientific, Waltham, MA, USA), and detected using chemiluminescence with Immobilon Forte Western HRP Substrate (WBLUF0500; Merck Millipore, Burlington, MA, USA).

### 4.6. Protein Expression Quantification

To quantify the protein amount, the soluble protein extract was boiled in SDS buffer as described above. To compare the protein concentration, 62.5 ng/12 μL, 250 ng/12 μL, and 1 μg/12 μL bovine serum albumin (BSA) solutions were prepared. Then, 12 μL samples and BSA solutions were loaded on an SDS-polyacrylamide gel. After electrophoresis, the gel was stained with Coomassie Brilliant Blue (CBB).

### 4.7. Image Analysis and Statistics

The leaf and stem areas with and without GFP expression were calculated using ImageJ 1.54g software (National Institutes of Health, Bethesda, MD, USA). The same software was used to compare the band intensities in the CBB-stained gels to determine the amount of protein expressed in the leaf tissues of the Enrei variety. For statistical analysis of the sample results, RStudio version 2022.07.2 + 576 (Posit, Boston, MA, USA) was used. Normally distributed data were presented as the mean ± standard deviation (SD). Analysis of variance (ANOVA) was performed and means were compared using Tukey’s test (*p* < 0.05).

## Figures and Tables

**Table 1:** Comparative analysis of green fluorescent protein (GFP) expression area in different soybean tissues among soybean varieties. ++, medium to high expression level (more than 50% infiltrated tissue expressed GFP signal); +, medium to low expression level (less than 50%); −, no GFP emission; GFP expression is compared with that of the negative control (no infection); *n* = 3.

	Tissue			
Variety	Leaf	Seedling	Cotyledon	Hypocotyl
*Glycine max* cv. Enrei	++	++	+	+
*Glycine max* cv. Fukuyutaka	+	+	−	−
*Glycine max* cv. Williams 82	+	+	+	+
*Glycine max* cv. Doko	+	+	−	+
*Glycine max* cv. Cristalina	+	+	−	+

## Data Availability

Data are contained within the article or Supplementary Materials.

## Supplementary Materials

The following supporting information can be downloaded at: https://www.mdpi.com/article/10.3390/plants13060858/s1. Figure S1: GFP expression samples from syringe infiltration. Figure S2: GFP expression dynamics in leaf samples following vacuum infiltration over time. Figure S3: Copy of the original Western blot picture.
