# Supplementary material for: Comparative Evaluation of Transient Protein Expression Efficiency in Tissues across Soybean Varieties Using the Tsukuba System
Source: Plants (Basel). 2024 Mar 16;13(6):858. doi: 10.3390/plants13060858 (PMC10975771; doi:10.3390/plants13060858)
Supplement: Supplementary file 1 [file plants-13-00858-s001.zip › Supplementary Materials.pdf]

Genotype	Leaf	
	Bright-field	GFP filter
pTKB3-eGFP	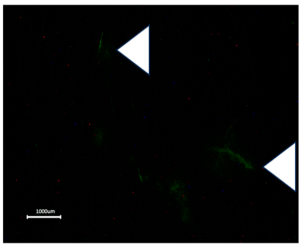	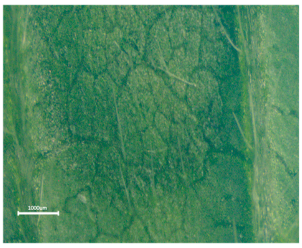
pTKB3-eGFP	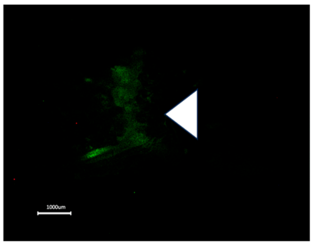	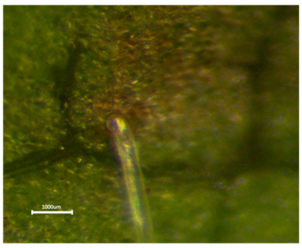
Control (pTKB3)	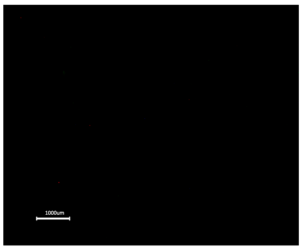	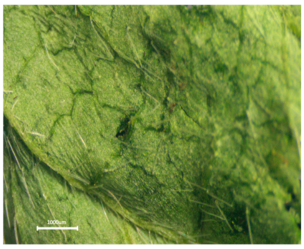

Figure S1. GFP expression samples from syringe infiltration. White arrows pointing the GFP expression. Bars representing 0,1cm.

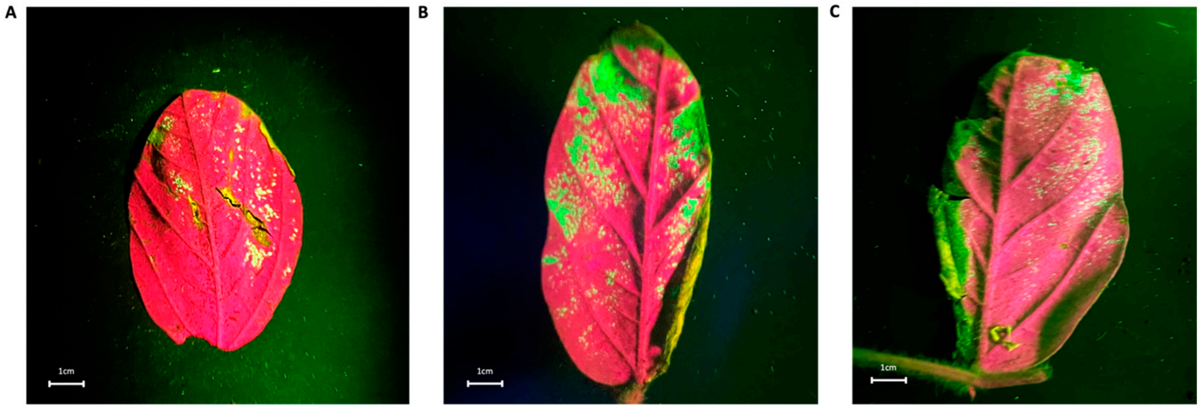

Figure S2. GFP expression dynamics in leaf samples following vacuum infiltration over time. (A) Leaf sample after 2 days of infiltration; (B) Leaf sample after 7 days of infiltration; (C) Leaf sample after 10 days of infiltration. Bars representing 1cm.

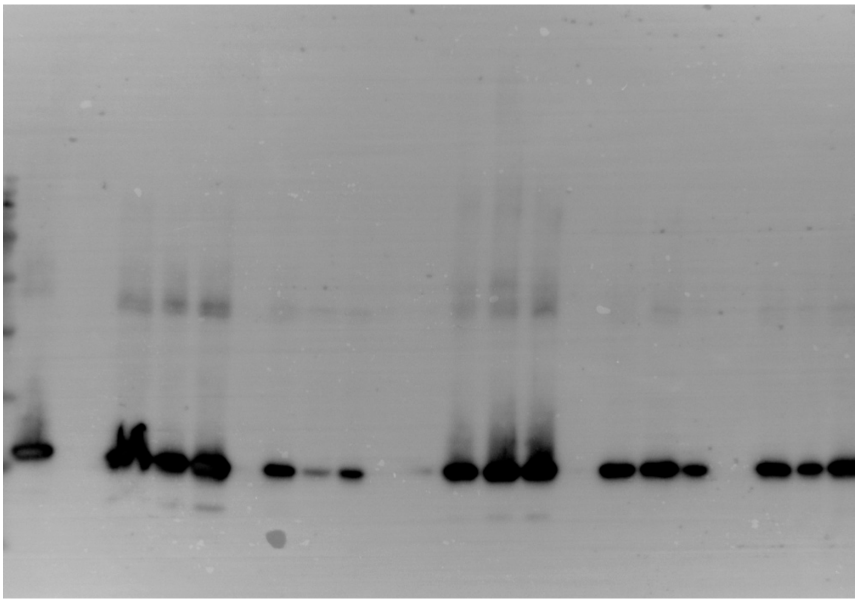

Figure S3. Copy of original western-blot picture.
