# Supplementary figures and images for: Comparative Evaluation of Transient Protein Expression Efficiency in Tissues across Soybean Varieties Using the Tsukuba System
Source: Plants (Basel). 2024 Mar 16;13(6):858. doi: 10.3390/plants13060858 (PMC10975771; doi:10.3390/plants13060858)

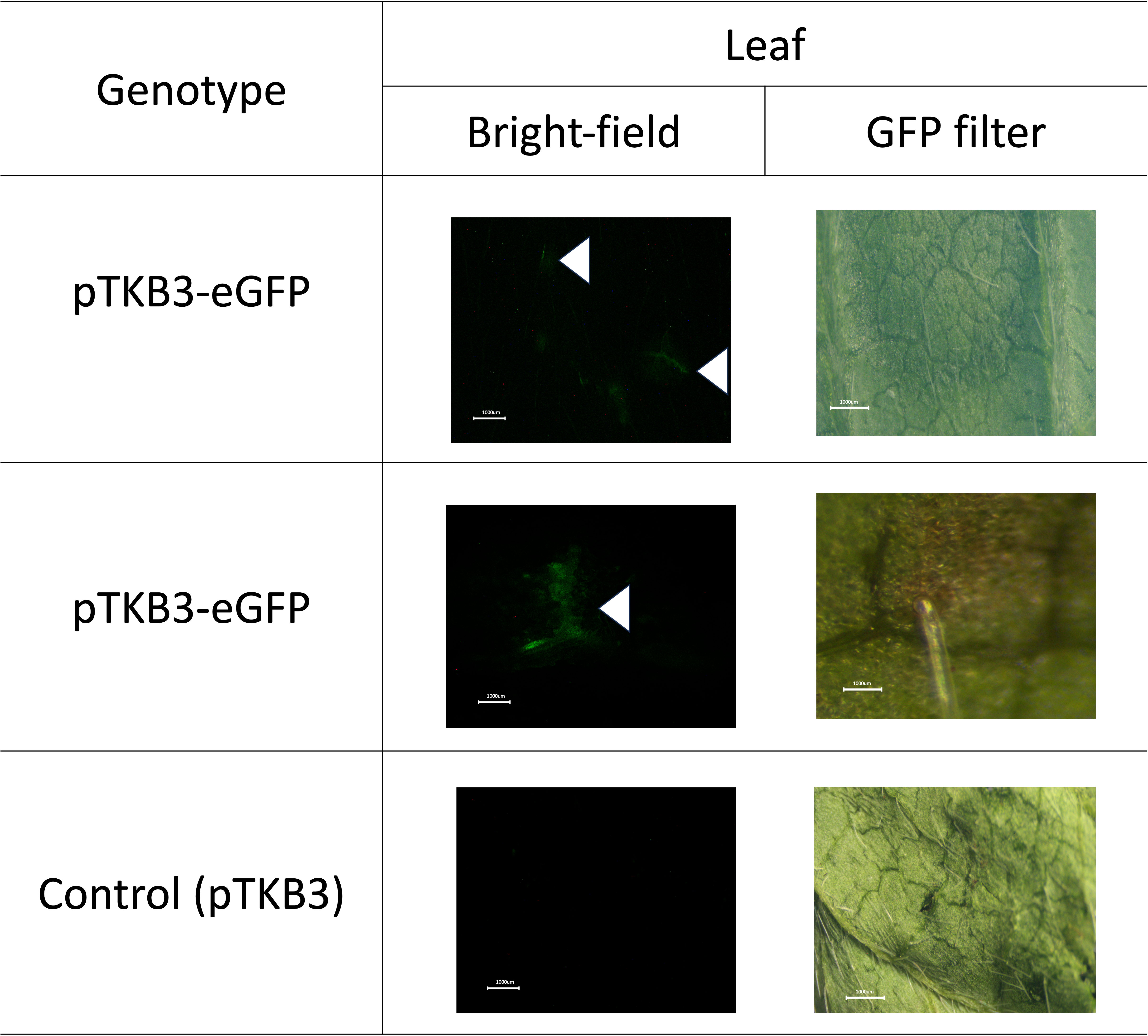

Supplement: Supplementary file 1 [file plants-13-00858-s001.zip › FigureS1.png]

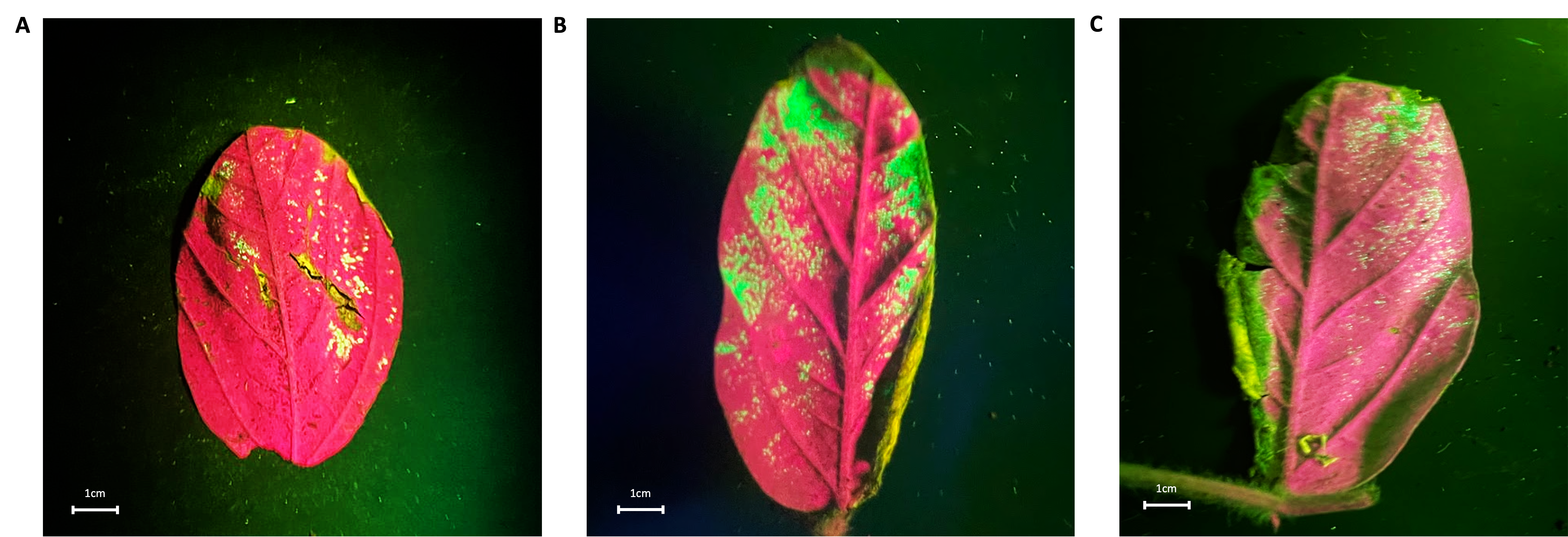

Supplement: Supplementary file 1 [file plants-13-00858-s001.zip › FigureS2.png]

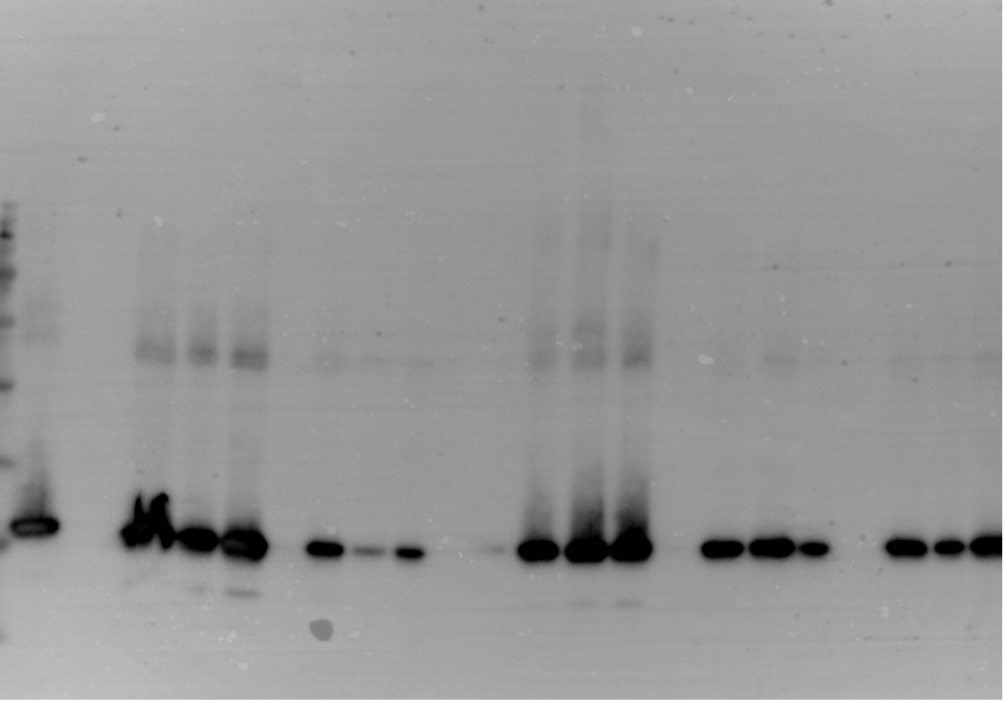

Supplement: Supplementary file 1 [file plants-13-00858-s001.zip › FiguresS3.jpg]
